# Hybrid-RViT: Hybridizing ResNet-50 and Vision Transformer for Enhanced Alzheimer’s disease detection

**DOI:** 10.1371/journal.pone.0318998

**Published:** 2025-02-14

**Authors:** Hongjie Yan, Vivens Mubonanyikuzo, Temitope Emmanuel Komolafe, Liang Zhou, Tao Wu, Nizhuan Wang

**Affiliations:** 1 Department of Neurology, Affiliated Lianyungang Hospital of Xuzhou Medical University, Lianyungang, China; 2 College of Health Science and Engineering, University of Shanghai for Science and Technology, Shanghai, China; 3 Collaborative Research Center, Shanghai University of Medicine & Health Sciences, Shanghai, China; 4 Department of Radiology, Jiading District Central Hospital Affiliated to Shanghai University of Medicine & Health Sciences, Shanghai, China; 5 Department of Chinese and Bilingual Studies, The Hong Kong Polytechnic University, Hung Hom, Kowloon, Hong Kong SAR, China; Gachon University, REPUBLIC OF KOREA

## Abstract

Alzheimer’s disease (AD) is a leading cause of disability worldwide. Early detection is critical for preventing progression and formulating effective treatment plans. This study aims to develop a novel deep learning (DL) model, Hybrid-RViT, to enhance the detection of AD. The proposed Hybrid-RViT model integrates the pre-trained convolutional neural network (ResNet-50) with the Vision Transformer (ViT) to classify brain MRI images across different stages of AD. The ResNet-50 adopted for transfer learning, facilitates inductive bias and feature extraction. Concurrently, ViT processes sequences of image patches to capture long-distance relationships via a self-attention mechanism, thereby functioning as a joint local-global feature extractor. The Hybrid-RViT model achieved a training accuracy of 97% and a testing accuracy of 95%, outperforming previous models. This demonstrates its potential efficacy in accurately identifying and classifying AD stages from brain MRI data. The Hybrid-RViT model, combining ResNet-50 and ViT, shows superior performance in AD detection, highlighting its potential as a valuable tool for medical professionals in interpreting and analyzing brain MRI images. This model could significantly improve early diagnosis and intervention strategies for AD.

## Introduction

Alzheimer’s disease (AD) is a brain ailment that gradually impairs memory, reasoning, and, eventually, the ability to do even the simplest tasks. Majority of individuals suffering from this illness experience symptoms that onset later in life, typically AD most commonly affects people over the age of 65 [1]. Age-related factors have been observed to increase the incidence of AD globally in last decades. AD is the most common cause of dementia and may contribute to 60–70% of cases, according to the World Health Organization (WHO) report, more than 55 million people have dementia worldwide [2].

AD was initially identified by Dr. Alzheimer in 1906. He observed changes in brain tissue among patients exhibiting symptoms such as memory loss, language problems, and unpredictable behavior. Through brain examinations, he discovered the presence of abnormal clumps known as amyloid plaques and tangled bundles of fibers referred to as neurofibrillary (tau) tangles. These amyloid plaques and tau tangles are still considered key characteristics of AD. Additionally, an important indicator involves the loss of connections between neurons in the brain [3, 4]. The initial symptoms of AD can vary from person to person, with cognitive impairment often being one of the first signs. However, difficulty in finding the right words, visual impairment or spatial awareness, and impaired reasoning or judgment are indicative of the early stages of this disease, it is important to recognize that other different facets of cognition may also serve as early warning signs. It is crucial to note that mild cognitive impairment (MCI) can be an early indicator of AD; but, not everyone with MCI will necessarily progress to the full-blown AD. In some cases, MCI may remain stable or even improve without progressing to AD [4, 5]. The stages of AD typically exhibit a progressive pattern, yet each individual traverses these stages in a unique manner. The stages of dementia, including the preclinical stage, mild stage, moderate stage, and severe stage [6, 7], are crucial for understanding AD. Understanding these stages help healthcare providers and family members to make informed decisions about the appropriate care for such individuals. In diagnosing AD, healthcare providers typically conduct interviews and use various tests to assess the individual’s cognitive function. Memory tests that function like puzzles or word games, are commonly adopted [8]. Additionally, the healthcare providers may collect health histories and conduct tests to eliminate other potential causes of memory loss or disorientation. These tests may include brain scans, such as Computed tomography (CT), Magnetic Resonance Imaging (MRI), or Positron Emission Tomography (PET) scans.

Artificial intelligence (AI), particularly machine learning, has been widely adopted in disease prediction, including mortality risk prediction in sepsis [9] and brain function analysis for conditions such as Alzheimer’s disease (AD) and dementia [10]. Recently, deep learning (DL)-based models have been used to predict treatment responses during Transarterial Chemoembolization (TACE) [11] and have also proven effective as diagnostic tools for AD by classifying patients into distinct diagnostic groups [12]. According to Jo et al. [12], these models, whether trained on separate datasets or combined datasets, significantly improve the accuracy of AD diagnosis. These models, whether utilizing separate datasets or combining them, significantly improve the accuracy of AD diagnosis. Furthermore, they demonstrate the ability to predict the progression of MCI subjects to AD. This predictive capability is essential for early intervention and the development of personalized treatment strategies for individuals at risk of developing AD. Convolutional Neural Networks (CNNs) are DL-based algorithms commonly used for image processing [13]. By adopting convolutional layers, CNNs automatically learn and extract hierarchical features from the input data. This facilitates effective pattern recognition, object detection, and classification, rendering CNNs widely applicable in computer vision tasks. CNNs have demonstrated outstanding performance in image classification, such as in study [14] adopted DenseNet-121 with a soft-attention block to analyze DaTscan images, achieving 99.2% accuracy in distinguishing Parkinson’s disease from normal cases by focusing on key brain regions, particularly the putamen and caudate, with superior performance compared to prior research, in [15]. This study explores the use of Surface Enhanced Raman Spectroscopy (SERS) combined with machine learning to accurately and rapidly differentiate Shigella species from Escherichia coli, overcoming the limitations of traditional methods, with Convolutional Neural Networks (CNN) demonstrating the best performance for bacterial discrimination, furthermore in [16] achieved high accuracy. Adequate training data is crucial for enhancing the generalization and accuracy of CNNs in recognizing and classifying objects. Unfortunately, owing to patient privacy concerns, there is a scarcity of publicly available image datasets for dementia, therefore challenges such as overfitting, exploding gradients, and class imbalance emerge prominently during the training of CNN-based models, significantly impacting their performance. To tackle these challenges, researchers have proposed different methods like in [17] introduces a federated epistasis detection framework (FedED-SegNAS) that combines privacy-preserving methods, fuzzy logic with CNNs, and optimized neural architecture to securely and efficiently analyze multi-institutional genomic data for disease risk identification. Another most commonly used methods are transfer learning methods based on CNNs [18–20].

Recently, Vision Transformers (ViT) has attracted interest in image processing due to their scalability and computational efficiency and the fact that they use the self-attention mechanism, which is also beneficial for image classification tasks [21]. ViT architecture for solving computer vision (CV) problems, based on the architecture of the transformer encoder. The main distinguishing features of this model are the partition of an image into disjoint patches, using positional embeddings to represent the sequence of patches in the image, and the use of the attention mechanism [22]. Additionally, ViT-based models have demonstrated superior performance compared to CNNs in the ImageNet dataset challenge [23, 24]. They have also excelled as state-of-the-art models on various other image datasets [25]. Despite the fact that ViT performs well in CV tasks, the structure of ViT models is very huge, and they require a huge amount of data for training. Besides, the computational requirements for training are higher than that of CNNs counterpart, which makes them rare applied in the medical field.

Most researchers primarily concentrated on utilizing pure CNNs [26–30] or ViT, as exemplified in the followings [31–34]. However, these studies encountered various challenges such as poor performance attributed to model bias, issues with feature extraction, limitations in dataset size, and issues related to exploding and vanishing gradient descent. To our best knowledge, there are still limited studies combining the CNN and ViT for medical image classification for AD. Inspired by the concept of transfer learning and the remarkable performance of ViT, as demonstrated in the work of Kadri et al. [35] where ViT was integrated with CNN, we propose a novel approach called Hybrid-RViT for improved Alzheimer’s Disease detection. This model combines ResNet-50 and Vision Transformer (ViT) to improve the accuracy and effectiveness in detecting AD. In this paper we cascaded pretrained ResNet -50 with ViT to perform image classification to predict different stages of AD.

Early detection of Alzheimer’s disease allows for timely interventions, improving patients’ quality of life, enabling families to plan for care, and facilitating access to effective treatments. It also promotes participation in research, benefiting both patients and caregivers. Existing studies often struggle with limited training data and rely on single models, such as CNNs or ViTs, which can have too many parameters and fail to explore potential synergies between models. Additionally, these studies usually lack comprehensive ablation analyses, making it difficult to assess the impact of specific components. This study aims to address these limitations by developing automated algorithms for predicting classes of AD using MRI medical imaging data. The goal is to create hybrid model that can assist in diagnosing the stages of AD disease based on the information extracted from MRI images.

This article contributes in the following ways:

1. The proposed Hybrid-RViT can be used to diagnose and predict the AD stages on the MRI images. We employ a CNN model to extract local features from brain MRI image data. To address the persistent challenge of limited medical image data for effective model learning, the proposed Hybrid-RViT leverages transfer learning to mitigate the impact of a small dataset.
2. We incorporate the ResNet model, known for its special ability to maintain feature identity during training, thereby addressing problems related to overfitting and vanishing gradients. Moreover, the ViT model’s application of the self-attention mechanism also enables the capturing of long-term dependencies.
3. We assess the performance of Hybrid-RViT model in predicting AD stages on MRI images. By comparing Hybrid-RViT with other models in terms of classification performance, Hybrid-RViT demonstrates favorable accuracy of 95% which makes it applicable for classification tasks within the medical field.

## Related works

The DL-based algorithms have found applications in the diagnosis and prediction of diseases. While CNN are commonly used for AD diagnosis, ViT has exhibited superior performance, especially in image classifications. The ability of ViT to capture direct correlations between images makes it potentially more effective in analyzing complex brain images compared to conventional CNN, considering the intricate network of the brain. In this section, we review research papers that explore the use of CNN and ViT in medical image processing, with a specific emphasis on image classification, streamline down the context of AD.

Shin et al. [33] introduced a novel approach utilizing ViT for the classification of dementia images obtained from PET scans. The ViT demonstrated superior performance compared to a convolutional neural network (CNN) model, VGG-19, in binary classification (normal vs. abnormal), its effectiveness in ternary classification (healthy control, mild cognitive impairment, and AD) was less pronounced. Consequently, the presumed superiority of ViT over CNN in AD classification remains inconclusive based on these findings. The utilization of additional datasets could facilitate a more comprehensive comparison and evaluation of the performance between CNN and ViT in binary classification and multiple classification tasks.

Xing et al. [36] presented a novel model for Alzheimer’s disease (AD) diagnosis, trained on multimodal Positron Emission Tomography (PET) images (PET-AV45 and PET-FDG). Deviating from conventional multimodal 3D/2D convolutional neural network (CNN) architectures, their design employs ViT as a substitute for CNN. To alleviate computational costs, they fused multimodal 2D images and fed them into a parallel ViT model for feature extraction, followed by classification for AD diagnosis. Their proposed model achieved an accuracy of 0.91 and an area under the curve (AUC) of 0.95 in their experiments. While the proposed model demonstrates promising results, its reliance on a limited dataset and lack of incorporation of advanced fine-tuning techniques raises concerns regarding its generalizability and potential for further improvement. Expanding the dataset and exploring sophisticated fine-tuning strategies could enhance the model’s accuracy and robustness.

Xin et al. [37] investigated the application of data augmentation techniques to expand training data for AD diagnosis using a ViT model. Despite the promising performance of ViT in computer vision tasks, the potential for overfitting remains, particularly with limited training data. To address this concern, the researchers employed various augmentation methods, including flip and rotation, cutmix, and mixup. The study emphasizes the significance of data augmentation in improving the performance of ViT-based models for AD diagnosis. The findings indicate that mixup augmentation demonstrates superior effectiveness, achieving the highest accuracy at 89.61%. By adopting the transfer learning mechanisms can improve the performance of the model

Carcagnì et al. [38] conducted the research, which aims to enhance the automatic detection of dementia in MRI brain data through computer-aided diagnosis (CAD). The study explores three deep CNN models (ResNet, DenseNet, and EfficientNet) and two transformer-based architectures (MAE and DeiT) to map input images to clinical diagnosis. The comparison results show transformer architectures, particularly DeiT, achieve the best classification results and display greater robustness against added noise from increased slices. The author concludes that the transformer shows promise of being used in real-world applications. While transformer architectures, particularly DeiT, have demonstrated superior performance in dementia diagnosis compared to CNNs, further research is needed to evaluate their generalizability and adaptability to real-world clinical settings. This includes conducting rigorous studies with large-scale, diverse datasets and thorough clinical validation. Additionally, an ablation study experiment would be beneficial to elucidate the factors contributing to the superior robustness of transformers compared to CNNs.

Zhang et al. [39] conducted a comparative analysis to assess the performance of VIT models in contrast to CNN-based models for classifying AD through MRI scans. The study also explores the applicability of a shallow 3D CNN-based model in this context. The findings reveal that the shallow 3D CNN-based model, ConvNet3D-4, attains satisfactory results in AD classification using MRI scans. The authors suggest that intricate CNN architectures may not be imperative for this particular task, and simpler models can achieve good performance. While Kushol et al. [40] demonstrated the potential of ViT models in AD detection, their exclusive reliance on global image features raises concerns about the method’s ability to capture fine-grained details crucial for accurate diagnosis. Additionally, the evaluation solely depends on the ADNI benchmark dataset which limits the generalizability of the findings. Incorporating local feature extracted by the CNNs and conducting more comprehensive evaluations on diverse datasets would be crucial to establish the true effectiveness of ViT-CNNs models in real-world clinical scenarios.

In this review, we’ve determined that the majority of studies rely on the utilization of either on pure conventional CNN or pure ViT models. Nevertheless, these investigations have highlighted certain limitations:

- The CNN models exhibit several drawbacks concerning feature representation and the necessity for extensive amounts of data. Additionally, they fall short in capturing and encoding long-range relationships at the pixel level within the input image.
- ViT has showcased commendable performance in CV and image classification tasks owing to the efficacy of the self-attention mechanism. Nonetheless, they demand a substantial amount of data for effective training.

Due to the limited research on the application of hybrid CNN and ViT models, there remains a need for further studies to explore their potential and effectiveness in various domains, the novelty of this study lies in addressing the aforementioned issues by introducing the DL model based on CNN and ViT. The CNN model achieves robust performance in extracting high-level features and generalizability, while the ViT model adeptly captures long-range dependencies, thereby complementing each other’s. Through the integration of these models, the proposed Hybrid-RViT model demonstrates noteworthy enhancements in performance accuracy and robustness.

## Methodology

### Dataset

In this study, we employed T1-weighted MRI scans from the Open Access Series of Imaging Studies (OASIS) dataset, The dataset consists of a cross-sectional collection of non-demented and demented images of subjects [41]. For each subject, 3 or 4 individual T1-weighted 2D MRI scans obtained in single scan sessions are included, in total 6400 images used in this study, the OASIS dataset serves as a valuable resource for researchers investigating aging and dementia. The study involved a sample of individuals comprising patients with Non-Cognitive Aging (NCA) and subjects diagnosed with Alzheimer’s Disease (AD) exhibiting various degrees of dementia, including very mild, mild, or moderate conditions, as determined through clinical examination. Table 1 summarizes the current dataset, which consists of subjects aged 60–96. At the time of their initial visit, 86 had a Clinical Dementia Rating (CDR) score of 0, indicating no dementia, while 64 had a CDR score greater than 0 (CDR 0.5, CDR 1, or CDR 2), indicating a diagnosis of very mild to moderate Alzheimer’s disease (AD).

**Table 1:** This table shows the subject characteristics of the dataset used in this study.

	Non demented					Demented				
Age group (years)	N	Mean	Male	Female	Convert	N	Mean	Male	Female	CDR 0.5/1
60–69	23	65.71	6	17	3	11	65.67	8	3	8/3^a^
70–79	35	74.91	11	24	4	36	73.97	20	16	29/7 ^a^
80–89	26	84.30	9	17	7	15	82.33	7	8	13/2 ^a^
90–99	2	92.50	0	2	0	2	93.00	1	1	1/1 ^b^

**Note**: N: Number of subjects; CDR: Clinical Dementia Rating.

^a^ Indicates severe dementia or impairment, defined as a CDR score of ≥ 3, while

^b^ indicates mild dementia or impairment, defined as a CDR score of 1.

### Image preprocessing

Data preprocessing is crucial for enhancing the performance of deep learning models. Python libraries such as open CV, Nibabel, Nilearn and Numpy are used for image preprocessing, encompassing tasks such as format conversion, image resizing, and conversion to an array. In order to reduce data-induced bias in the model, the dataset is partitioned into 20% for testing, 70% for training, and 10% for validation. In accordance with the model building, classification was performed, and the model was evaluated using various metrics. To prevent data leakage during data splitting and to address the lack of an independent test set, we used Nipype, an open-source Python project. Nipype provides a uniform environment that facilitates seamless interaction between various neuroimaging software tools and algorithms, regardless of their programming language, within a single workflow.

### ResNet-50 architecture

The ResNet-50 is a deep learning neural network architecture from the ResNet family, introduced by Kaiming [42] to address the challenge of optimizing deep architectures where performance may decline as the network goes deeper. It incorporates residual learning.

A residual block can be defined as:

F(I)=H(I)+I (1)

where *H*(*I*) is the learned residual mapping, *F*(*I*) is the desired output, and *I* is the input to the block. The network then learns the residual *H*(*I*) instead of learning *F*(*I*) directly. The ResNet-50 is a DL architecture designed for image classification tasks. It starts with an input image with 224×224 pixels and three colour channels. The initial convolutional layer, Conv, is used to perform feature extraction, followed by a 3×3 max-pooling layer, MaxPool. The core consists of 34 residual blocks, each with two convolutional layers and a shortcut connection, enhancing gradient flow during training. The final layer is a classification head, a global average pooling layer, and a fully connected layer. The algorithm in S1 Table shows the architecture of ResNet-50 layers, a deep neural network for image classification.

### Visual Transformer

The Visual Transformer (ViT), introduced in 2020 by Dosovitskiy et al. [21], represents images as sequences of tokens, similar to text, which are then processed and classified using a standard transformer architecture. Architecture is designed for image analysis and follows a structured process. It begins with the input to the ViT model, which is an image divided into a grid of non-overlapping patches. The input to the ViT model is an image divided into a grid of non-overlapping patches. Each patch has dimensions Input P*P*3, where, *P* is typically set to 16, and 3 represents the RGB color channels. The input patches are linearly embedded into a sequence of vectors, input: *X*^(0)^∈ℝ^N*P*3^, output *X*^(1)^∈ℝ^N*D^ where, *X* is input, *N* is the number of patches, *D* is the embedding dimension. The next phase is the position embedding represented as:

$$X^{\left(2\right)}=X^{\left(1\right)}+PE(X^{\left(1\right)})$$ (2)

*PE* represents positional encoding. The core of VIT consists of multi-head self-attention X∈ℝ^N*seq_leng*embed_dim^, and the linear projection by three parameters which are mathematical represented as:

$${Query:\;Q}_{h}={X*w}_{Qh},$$ (3)

$$keys{:\;K}_{h}={X*w}_{Kh},$$ (4)

$$Values:\;V_{h}={X*w}_{Vh}$$ (5)

*w*_*Qh*_, *w*_*kh*_, *w*_*Vh*_ are learnable weight matrices for each head. The scaled dot-product attention is mathematical represented as:

$$A_{h}=softmax\left(\frac{Q_{h}K_{h}^{T}}{\sqrt{d_{k}}}\right)$$ (6)

Where the softmax function computes the softmax function along the sequence dimension and *d*_*k*_ is the dimension of the key vectors (*d*_*k*_ = embed_dim/h). The outputs from all heads were concatenated, which mathematical is represented as:

$$Z_{h}conc(A_{1},A_{2,\ldots \ldots \ldots \ldots ..}A_{h})W_{o}$$ (7)

where *W*_*o*_ is a learnable weight matrix for output projection.


$$Output=Z_{h}+X$$ (8)

**Fig 1:**
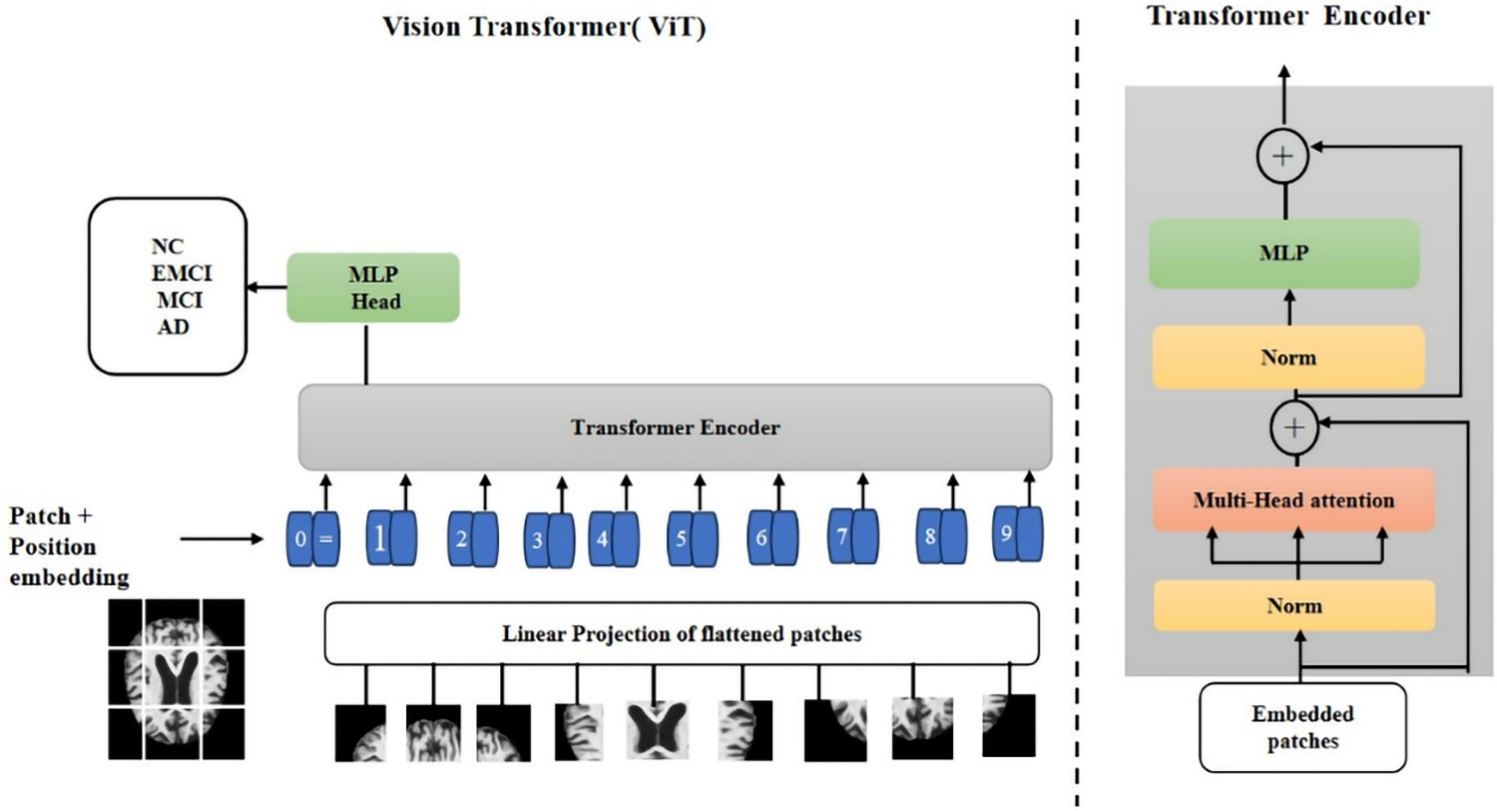
Illustration of a Vision Transformer (ViT): A neural network architecture designed for computer vision tasks. The diagram depicts the distinctive structure of the vision transformer, emphasizing its attention mechanisms and positional encoding techniques, facilitating efficient image data processing within a transformer-based framework.

The high-level representation of the ViT can be broken down into several steps as shown in Fig 1.

### Proposed Hybrid-RViT model

**Fig 2:**
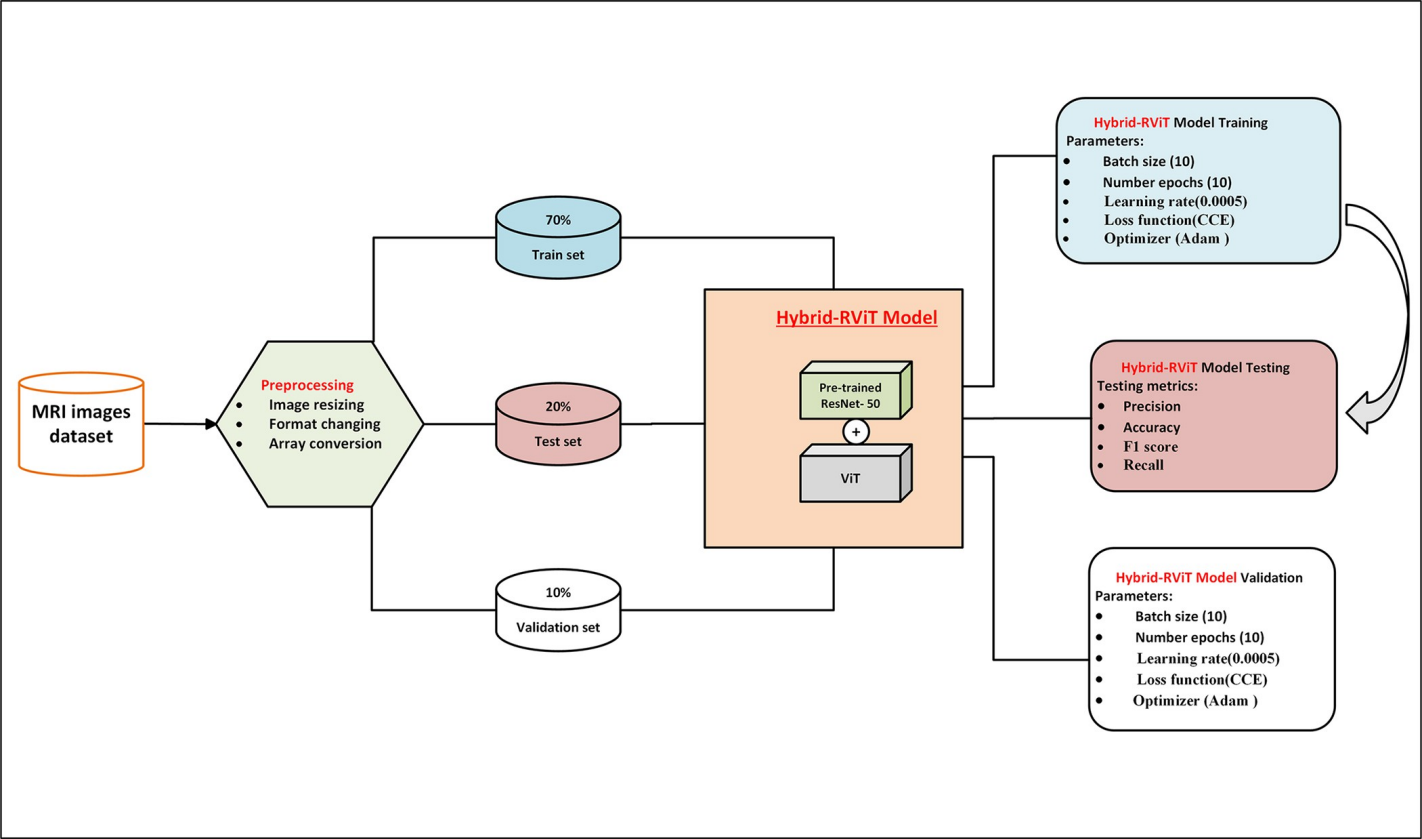
The workflow of the proposed Hybrid-RViT model.

**Fig 3:**
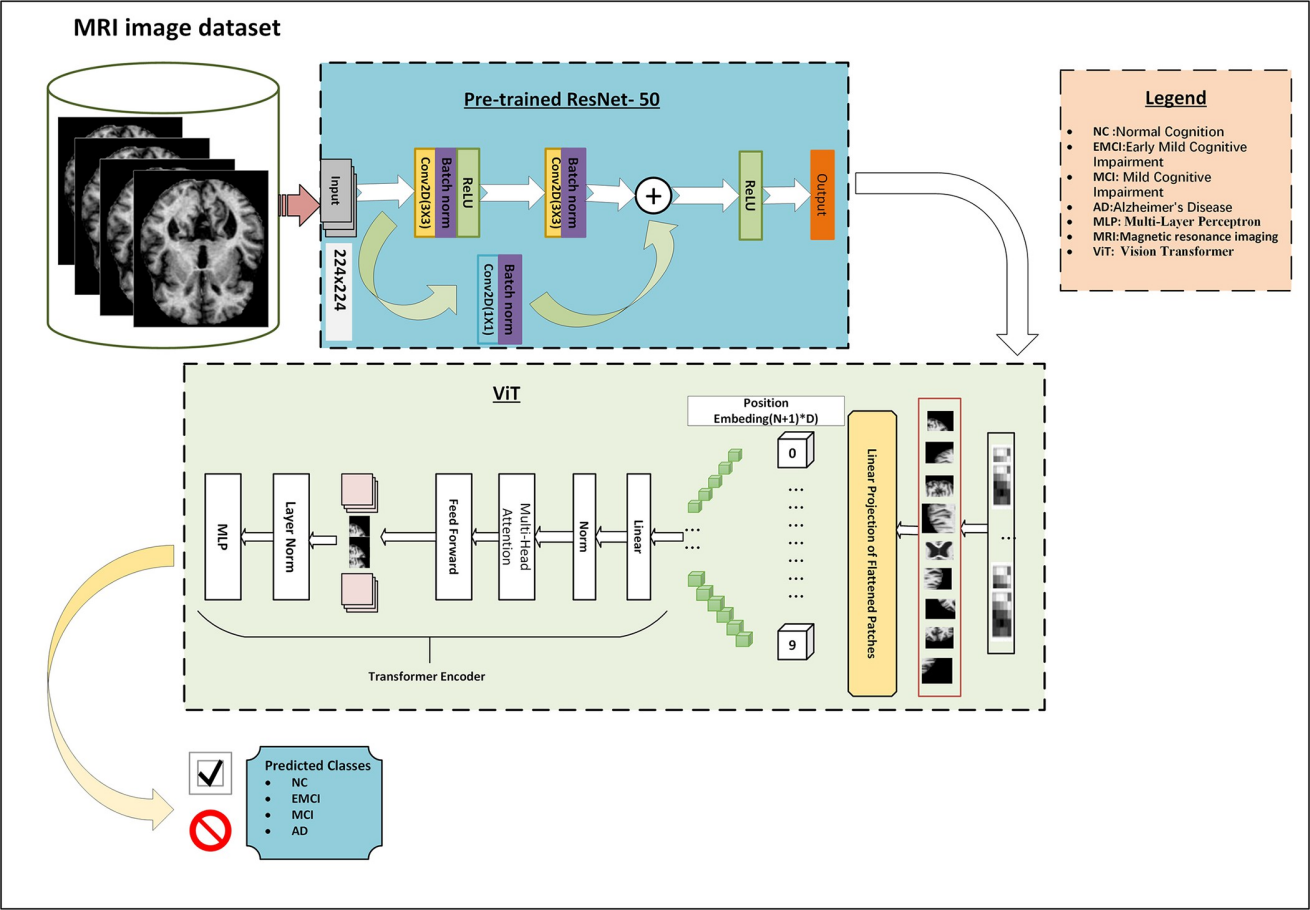
Overview of the Hybrid-RViT architecture. The process begins with inputting images of dimensions 224×224, which are fed to ResNet-50 for feature extraction, the extracted features undergo processing through ViT in the form of patches (N+1)+D). A transformer encoder is then applied to perform self-attention. Finally, the learned features are passed through an MLP classifier for classification. The model optimization during training is performed using the validation set, while the test dataset is utilized to evaluate the model’s performance on unseen data.

The architecture of the proposed model consists of an input layer for images with dimensions, a pre-trained ResNet-50 backbone for processing images, patch embeddings for convolution, batch normalization, and reshape operations, and positional embeddings for capturing positional information, the model combines these embeddings through element-wise addition, resulting in a tensor with dimensions. A class token is introduced to incorporate class information. The transformer encoder loop refines the embeddings iteratively, applying multi-head self-attention and feed-forward neural networks. Layer normalization is applied, and a slicing operation retains the first position, corresponding to the class token, resulting in a tensor with dimensions The output layer computes classification logits using learned weights and biases, enabling the model to make predictions for various classification tasks. The work flow and proposed Hybrid-RViT Model is presented in the Figs 2 and 3 respectively. The proposed Hybrid-RViT Model is presented in the following pseudocode, S2 Table.

## Experimental result presentation

In this section, we provide a description of the measures employed to evaluate the performance of the proposed model. Furthermore, the proposed approach is compared with a few several other current approaches. The experiments evaluated in this work were conducted on a brain MRI data set for AD detection. The experiments were implemented on a Google Colab, a cloud-based platform inspired by Japyter Notebook. All networks are trained by using a batch size of 16. The Adam optimization algorithm [43] is used in this experiment instead of the classical stochastic gradient descent optimizer to update weights based on training data. The Adam optimizer is applied with a learning rate of 5×10^−5^. Table 2 summarizes the hyper parameters used during model training.

**Table 2:** Hyper-parameters used during training of proposed Hybrid-RViT.

Hyperparameters	Values
**Learning rate**	0.00005
**Optimizer**	Adam
**Loss function**	Categorical Crossentropy
**Batch size**	16
**Number of epochs**	10

**Fig 4:**
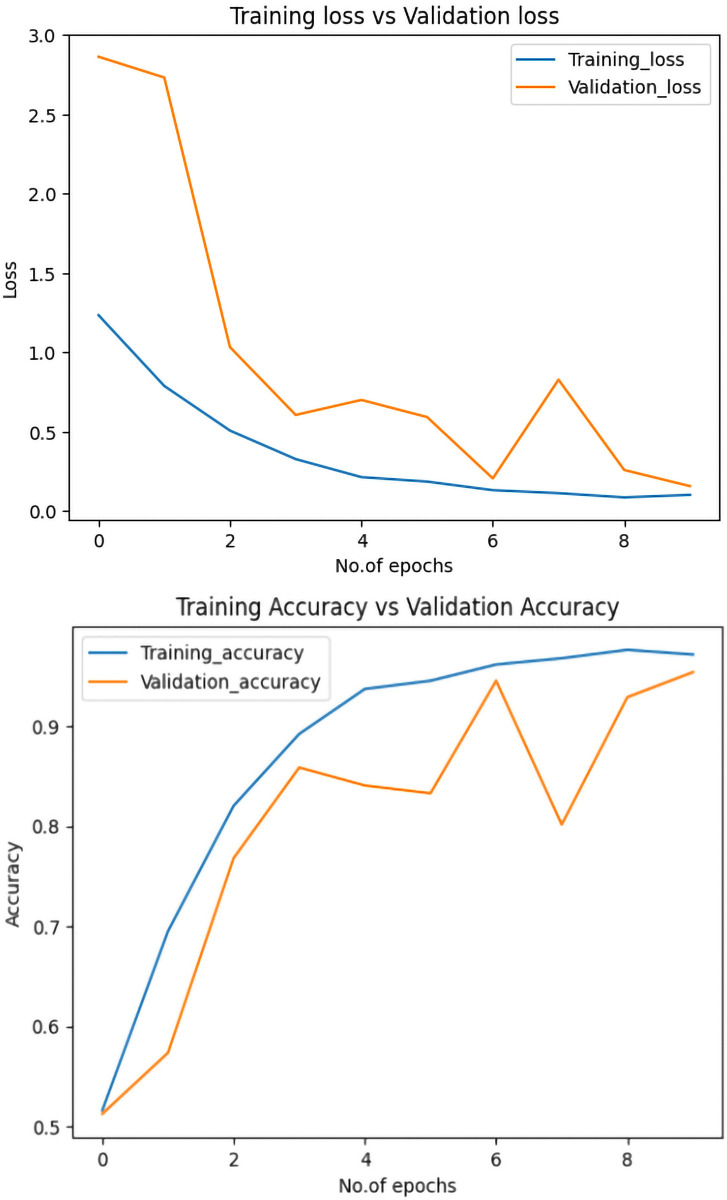
Plot of training accuracy and loss. Fig 4A displays training accuracy and validation accuracy, while Fig 4B shows training loss and validation loss.

Fig 4 shows the proposed model has achieved 97% accuracy during training and 94% accuracy on the validation dataset. Also, the training loss and the validation loss regard the progress of the number of epochs, with the final loss equal to 0.10 and validation loss equal to 0.15. Since the margin between training accuracy and validation accuracy is not large, the fact that both the loss and validation loss exhibit the same pattern indicates that the model doesn’t suffer from overfitting or underfitting issues on the OASIS dataset.

### Evaluation metrics

The classification report provides a comprehensive assessment of the model’s performance for each class and overall, in this study various metrics used are precision, recall, and f1-score, accuracy. Precision is the ratio of correctly predicted positive observations (true positives) to the total predicted positives (TP: true positives + FP: false positives). It indicates how well the model is performing when it predicts a positive class, higher precision implies fewer false positives.


$$Precision=\frac{TP}{TP+FP}$$ (9)

Recall is the ratio of correctly predicted positive observations to the total actual positives (TP: true positives + FN: false negatives). It measures how well the model is capturing the positive instances, higher recall connotes fewer false negatives.


$$Recall=\frac{TP}{TP+FN}$$ (10)

F1-score is the harmonic mean of precision and recall. It provides a balance between precision and recall and is useful when there is an uneven class distribution. High F1-score indicates that the model is performing well in terms of both precision and recall.


$$F1=\frac{2TP}{2TP+FP+FN}$$ (11)

The accuracy is the overall percentage of correct predictions made by the model across all classes.


$$Accuracy=\frac{\left(TP+TN\right)}{TP+TN+FN+FP}$$ (12)

Among the four classes, NC has highest precision of 98%, EMCI highest recall of 100%, and NC and EMCI has highest F1 score of 96%. Table 3 shows the classification reports of model on classification during training.

**Table 3:** Evaluation metrics of the Hybrid-RViT.

Classes	Precision	Recall	F1-score
NC	0.9750±0.0100	0.9300±0.0866	0.9500±0.0464
EMCI	0.9525±0.0619	0.7875±0.0626	0.8575±0.0303
MCI	0.8950±0.1150	0.9725±0.0083	0.9300±0.0704
AD	0.9275±0.0795	0.9450±0.0166	0.9375±0.0507

Note: NC: Normal Cognition, EMCI: Early Mild Cognitive Impairment: Mild Cognitive Impairment, AD: Alzheimer’s Disease.

**Fig 5:**
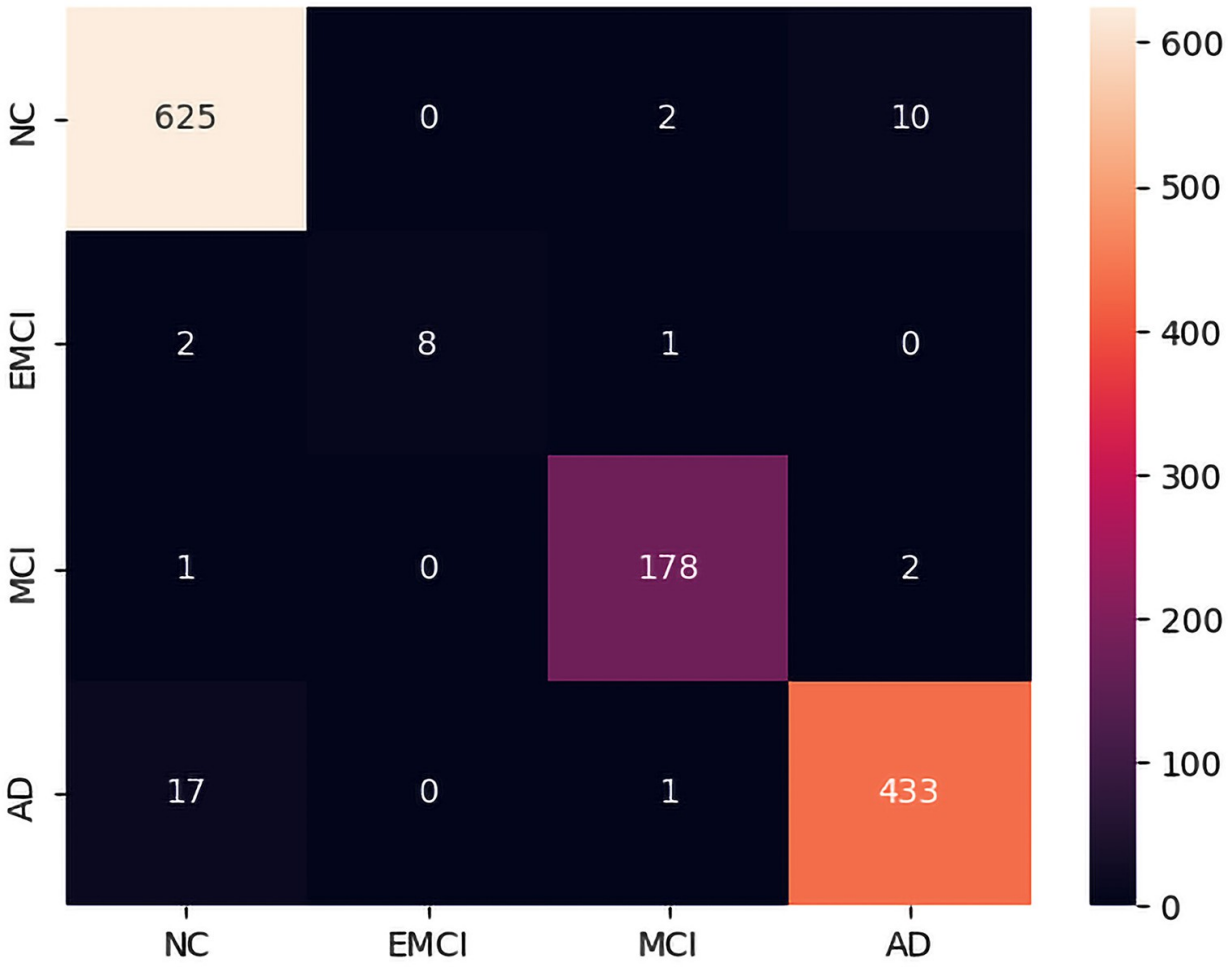
Confusion matrix for the Hybrid-RViT model on test data set.

In addition, we used a confusion matrix delineates the accurate classification of images into their respective categories, revealing 625 instances correctly attributed to the NC class, 8 to EMCI, 178 to MCI, and 433 to AD. Nevertheless, instances of misclassification transpired across categories, notably including 2 NC images erroneously categorized as EMCI and 10 as MCI, along with 2 EMCI images erroneously classified as NC and 1 as MCI. Additionally, 1 MCI image was inaccurately designated as NC and 2 as AD, while 17 AD images were misclassified as NC and 1 as MCI, denoted by the non-zero entries in the off-diagonal elements of the matrix, as shown in Fig 5. We present some samples of the images classified by the Hybrid-RViT model. While it correctly classifies most images from the dataset, some are wrongly predicted. This is because of the small dataset used during model training, which can hinder accurate classification of certain classes, as the model may struggle to discern subtle distinctions without sufficient examples. Moreover, proper preprocessing techniques raise the image’s quality so that the model can analyze it more effectively. Therefore, careful attention to both dataset size and preprocessing strategies is crucial to ensure robust classification performance across all classes.

## Discussion

**Fig 6:**
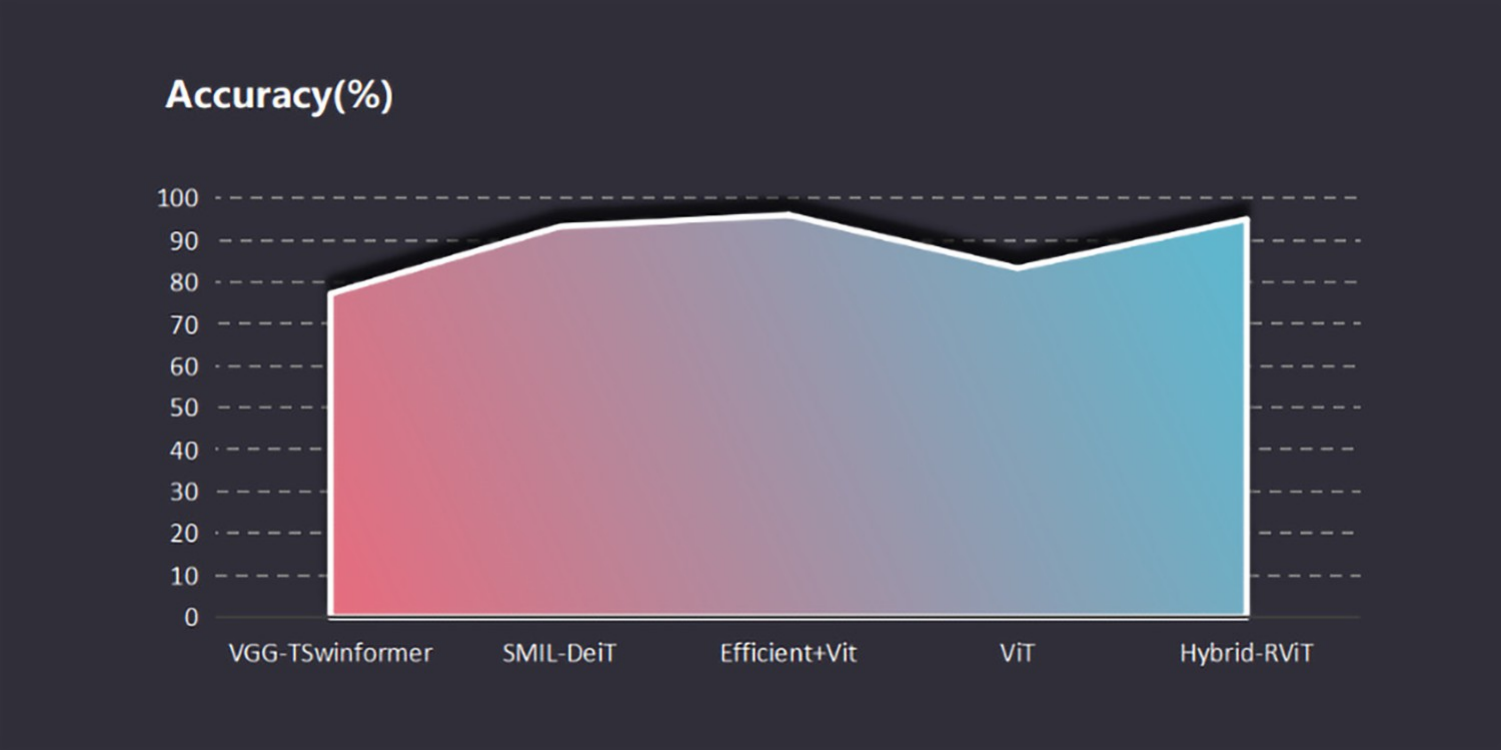
Comparative analysis of accuracy for proposed Hybrid-RViT model against VGG-TSwift, SMIL-DeiT, Efficient+ViT, and ViT.

The introduction of a novel deep learning hybrid model proposed in this study marks a significant advancement in the field of Alzheimer’s disease (AD) detection. The proposed Hybrid-RViT model integrates state-of-the-art architectural innovations tailored specifically for analyzing neuroimaging data associated with AD pathology, as shown in Fig 6, the accuracy of Hybrid-RViT on the test dataset is 95%. Our model exhibits better performance compared to recent state-of-the-art (SOTA) hybrid models such as VGG-TSwinformer [44] and SMILDEIT [45], as well as ViT [31]. The proposed model also outperforms other models that use MRI images along with well-known deep 2D CNN architectures. For instance, the VGG16 model achieved 64.3%, and ResNet-50 obtained 67.1% [46] in terms of accuracy. Furthermore, in [47], a novel deep-learning-based model for classifying healthy controls (HCs) and patients with Alzheimer’s disease (PwAD) based on eye-movement data is presented. Regarding AD recognition, their model achieves an average accuracy of 85%. In [48], the author achieves a classification accuracy of over 80% for the diagnosis of AD by combining information from the results of neuropsychological tests, diagnoses, and other clinical data with imaging features extracted solely via data-driven decomposition of MRI.

In the study [35], two modalities (PET+MRI) were employed in training the model, with Generative Adversarial Network (GAN) used as a method to increase the size of the dataset. These factors could have made their model more robust than ours. However, we can infer that our proposed model exhibits good performance for the following reasons: the pre-trained ResNet-50 model adopted as the backbone incorporates a technique called "skip connection" to address the vanishing gradient problem. This technique involves creating shortcuts that facilitate more effective gradient flow during training, enabling ResNet to successfully train deep networks. Additionally, the model employs ViT, which generates a sequence by dividing an image into numerous smaller sections. To capture the attention between patches, multi-head self-attentions are then applied to the sequence.

**Fig 7:**
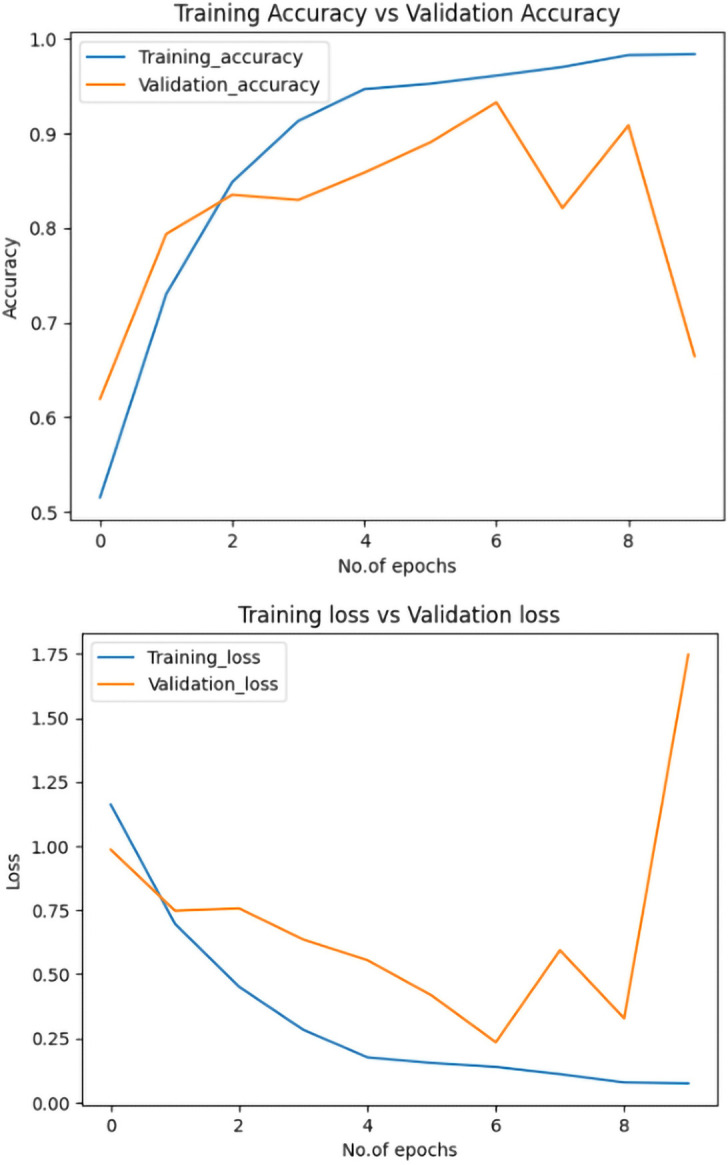
Results of the ablation study comparing ResNet-50 versus ResNet-101. Fig 7A shows the training accuracy and validation accuracy of Hybrid-RViT after replacing ResNet-50 with ResNet-101. In Fig 7B, the training loss and validation loss of Hybrid-RViT are shown after replacing ResNet-50 with ResNet-101.

For the ablation study aimed at exploring the contribution of the pretrained CNN model component in our proposed Hybrid-RViT model, we replaced ResNet-50 with the pretrained ResNet-101, which comprises large residual networks with 101 layers. The remaining model parameters were kept unchanged, and training was conducted under identical conditions, with the hyperparameters set to the same values and using the same dataset. The ablation result is shown in Fig 7, it revealed that after adopting ResNet with 101 layers, the model experienced the issue of overfitting. One of the reasons for this was that the model became overly complex, fitting noise in the training data rather than capturing the useful underlying patterns. One of the major strengths of the Hybrid-RViT lies in its ability to incorporate attention mechanisms and spatial invariance. By leveraging local connectivity, the model provides clinicians with insights into the specific regions and features contributing to its predictions.

Future research should prioritize validating the generalizability of the Hybrid-RViT model across diverse populations and healthcare settings. The model’s innovative architecture, combining ResNet-50 and Vision Transformer (ViT), provides significant advancements in neuroimaging analysis for Alzheimer’s disease (AD) detection. These architectural innovations offer deeper insights into critical features and regions contributing to predictions, providing clinicians with a reliable tool for early detection.

The implications of these findings are profound for the clinical management of AD. By accurately identifying AD patients, even in the early stages, the Hybrid-RViT can support early intervention strategies, which are important for slowing disease progression and ultimately improving patient outcomes. Furthermore, its computational efficiency and ability to process medical images effectively make it a promising candidate for integration into clinical workflows, particularly in resource-constrained scenarios. The adoption of Hybrid-RViT in clinical practice could significantly enhance diagnostic accuracy and enable better resource allocation for AD treatment.

Additionally, integrating multimodal data sources, such as genetic and clinical biomarkers, could enhance the model’s predictive capabilities and deepen our understanding of AD stages. Collaborative efforts to establish standardized protocols for data collection, model evaluation, and clinical validation will foster transparency and reproducibility, advancing the field of AD research and diagnostics.

Despite its potential, this study has some limitations. The performance of the Hybrid-RViT model was evaluated using a single dataset, and its ability to generalize to other datasets remains untested. To establish its real-world applicability, further validation on diverse datasets, particularly those from different hospitals and healthcare systems, is necessary.

Moreover, the model was trained using publicly available GPUs, which may not reflect the computational resources available in all clinical settings. The computational complexity and resource requirements of Hybrid-RViT could pose challenges for scalability and practical deployment in resource-limited environments.

The ablation study also revealed potential issues with overfitting when ResNet-101 was used as the backbone, highlighting the need for careful optimization of model complexity to prevent fitting noise instead of meaningful patterns in the training data.

Future research should address these limitations by exploring ways to enhance the model’s robustness, scalability, and generalizability. Collaborative studies incorporating diverse datasets and real-world scenarios will be critical for ensuring the Hybrid-RViT’s effectiveness across varied populations and clinical settings.

## Conclusion

In this study, we presented a novel Hybrid-RViT model for the automatic classification of AD using MRI images. Our experimental results demonstrate that the proposed model can effectively distinguish AD from healthy controls, even when trained on a relatively small dataset. However, the performance of the model may be limited by the dataset size and its ability to generalize to other populations or data sources. Future research will focus on addressing these limitations by testing the model on larger and more diverse datasets, particularly from multiple hospitals. Additionally, there is potential to enhance the robustness of the model by improving preprocessing techniques, tuning hyperparameters, and incorporating data augmentation strategies. Another direction for future work includes adapting the model for deployment on smaller devices for real-time, portable detection, and exploring the integration of both MRI images and clinical data to improve early detection of the progression from Mild Cognitive Impairment (MCI) to Alzheimer’s disease.

## Supporting information

S1 Table The algorithm shows the architecture of ResNet-50 layers, a deep neural network for image classification. (DOCX)

S2 Table The proposed Hybrid-RViT model is presented in the following pseudocode. (DOCX)
