# Supplementary material for: Hybrid-RViT: Hybridizing ResNet-50 and Vision Transformer for Enhanced Alzheimer’s disease detection
Source: PLoS One. 2025 Feb 14;20(2):e0318998. doi: 10.1371/journal.pone.0318998 (PMC11828341; doi:10.1371/journal.pone.0318998)
Supplement: S1 Table — (DOCX) [file pone.0318998.s001.docx]

| ***Layers*** | ***Algorithm*** |
| --- | --- |
| ***Step1: Input*** | $X^{(0)}\in\mathbb{R}^{224*224*3}$ |
| ***Step2: Conv*** | $X^{(1)}={Conv （X^{(0)}）Where X^{(1)}\mathbb{R}}^{112*112*64}$ |
| ***Step3: Maxpool*** | $input :X^{(1)}\in\mathbb{R}^{112*112*64}$  ${output :X}^{(2)}=MaxPool\left( X^{\left( 1 \right)} \right) Where X^{(2)}\in\mathbb{R}^{56*256*64}$ |
| ***Step4: ResBlock*** | ${input :X}^{\left( i \right)} Where i ,i=2\to35$  ${output :X}^{\left( i+1 \right)}=ResBlock(X^{\left( i \right)}),for i=2,3,\ldots..,35$  *For n Conv,* $n\in\mathbb{R}^{+}$*,*  $F^{(i)}=Conv2D(X^{\left( i \right)},W^{\left( i \right)},b^{\left( i \right)})$*, Conv operation*  $X^{\left( i+1 \right)}=F^{(i)}+X^{\left( i \right)},short connection$ |
|  |  |
| ***Step5: Global average*** | ${input :X}^{\left( 35 \right)}$  $output:X^{\left( 36 \right)}=G\left( X^{\left( 35 \right)} \right) WhereX^{\left( 36 \right)}\in\mathbb{R}^{2048}$ |
| ***Step6: Fully connected*** | ${input:X}^{\left( 36 \right)}$  ${ouput:Y=F(X}^{\left( 36 \right)},W_{F,} b_{F,})Where Y: final classification probabilities$ |

**S1 Table. The algorithm shows the architecture of ResNet-50 layers, a deep neural network for image classification.**
