# Supplementary material for: Hybrid-RViT: Hybridizing ResNet-50 and Vision Transformer for Enhanced Alzheimer’s disease detection
Source: PLoS One. 2025 Feb 14;20(2):e0318998. doi: 10.1371/journal.pone.0318998 (PMC11828341; doi:10.1371/journal.pone.0318998)
Supplement: S2 Table — (DOCX) [file pone.0318998.s002.docx]

**S2 Table. The proposed Hybrid-RViT Model is presented in the following pseudocode.**

| *Step1* | *start* |
| --- | --- |
| *Step2* | *# Input: Image*  Input MRI image |
| *Step3* | *# Combine features from ResNet-50 and ViT*  combined_features =concat (resnet_features, vit_features) |
| *Step4* | *# element-wise addition*  elem_result = fusion(combined_features) |
| *Step5* | *# Apply a classification head*  logits = classification_head(elem_result) |
| *Step6* | *# Output: Final prediction*  output = argmax(logits) |
| *Step7* | *return output* |
